# Sex aggregation and species segregation cues in swarming mosquitoes: role of ground visual markers

**DOI:** 10.1186/s13071-019-3845-5

**Published:** 2019-12-16

**Authors:** Serge B. Poda, Charles Nignan, Olivier Gnankiné, Roch K. Dabiré, Abdoulaye Diabaté, Olivier Roux

**Affiliations:** 1 Institut de Recherche en Sciences de la Santé (IRSS), Bobo-Dioulasso, Burkina Faso; 2 Laboratoire d’Entomologie Fondamentale et Appliquée, Unité de Formation et de Recherche en Sciences de la Vie et de la Terre (UFR-SVT), Université Ouaga I Pr. Joseph KI-ZERBO, Ouagadougou, Burkina Faso; 3 MIVEGEC, IRD, CNRS, University of Montpellier, Montpellier, France

**Keywords:** *Anopheles*, Mating, Sexual encounter, Speciation, Swarming behavior, Visual cues

## Abstract

**Background:**

Mating swarm segregation in closely related insect species may contribute to reproductive isolation. Visual markers are used for swarm formation; however, it is unknown whether they play a key role in swarm location, species segregation and sex aggregation.

**Methods:**

Using two sympatric closely related species of the *Anopheles gambiae* complex, *An. coluzzii* and *An. gambiae* (*s.s.*), we investigated in both laboratory and semi-field conditions (i) whether males of the two species use visual markers (black cloths) to locate their swarm; and (ii) whether the presence/absence and size of the marker may differentially affect swarm characteristics. We also investigated whether conspecific virgin females use these markers to join male swarm sites.

**Results:**

We showed that males of the two species used visual markers but in different ways: *An. coluzzii* swarm right above the marker whereas *An. gambiae* (*s.s.*) locate their swarm at a constant distance of 76.4 ± 0.6 cm from a 20 × 20 cm marker in the laboratory setup and at 206 ± 6 cm from a 60 × 60 cm marker in the semi-field setup. Although increased marker size recruited more mosquitoes and consequently increased the swarm size in the two species, *An. coluzzii* swarms flew higher and were stretched both vertically and horizontally, while *An. gambiae* (*s.s.*) swarms were only stretched horizontally. Virgin females displayed a swarm-like behavior with similar characteristics to their conspecific males.

**Conclusions:**

Our results provided experimental evidence that both *An. coluzzii* and *An. gambiae* (*s.s*.) males use ground visual markers to form and locate their swarm at species-specific locations. Moreover, the marker size differentially affected swarm characteristics in the two species. Our results also showed that virgin females displayed a swarm-like behavior. However, these “swarms” could be due to the absence of males in our experimental conditions. Nevertheless, the fact that females displayed these “swarms” with the same characteristics as their respective males provided evidence that visual markers are used by the two sexes to join mating spots. Altogether, this suggests that visual markers and the way species and sexes use them could be key cues in species segregation, swarm location and recognition.
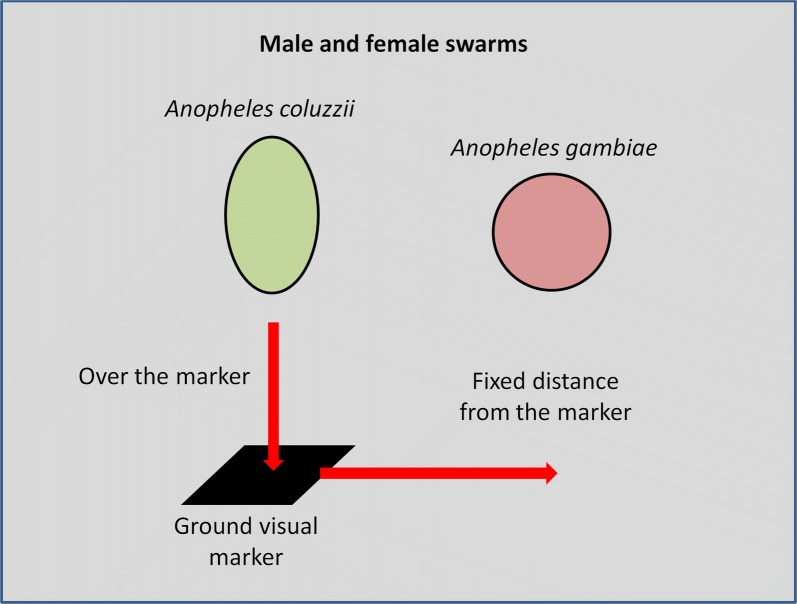

## Background

Animals sometimes gather in groups. This behavior can be a response to environmental heterogeneities or needs for social interactions [1–3]. They occur around an attractive resource and the individuals might disperse if this resource wanes or once the environmental heterogeneity is removed [3]. Aggregation of individuals can provide various advantages such as reduction of predation risk and more mating opportunities [1–3]. In some cases, gathering may contribute to the reproductive isolation [3, 4] of species with closely related species distinguishing themselves from each other thanks to species-specific aggregation preferences [4–7]. Actually, species segregation can occur either in space through the preference for different aggregation sites or over time with aggregations occurring at a same location but at different times [3, 5, 8–11].

In insects, aggregation for mating at a specific site is mainly observed in species that mate in flight [4–6, 8–10]. Males gather in swarms near female emergence, feeding, oviposition or resting sites [12–14], or specific locations without any relation with resources, where females come to choose a male (i.e. leks) [6, 15]. Swarming males usually gather in continuous flight over a landmark called “swarm marker” that can be any visual contrasting color or shape in the environment [16]. These markers have been described to be critical for swarm formation and to have an impact on swarm characteristics since the number of males and the swarm shape seem to be related to the marker size [8, 16–18]. Females are known to enter swarms and to acquire a mate in flight. However, in some species such as the dance-fly *Empis borealis* L. (Diptera: Empididae), females have been described to be the swarming sex [19] but these sex-role reversions seem to be rare and have been interpreted as behavioral “mistakes” (marker confusion by females) or a by-product of other aggregative behaviors (feeding or oviposition) [20]. Nevertheless, in some species in which the swarming sex is known to be the males, it has been described that in the absence of males, females produce a swarm-like behavior over markers, as is the case for crane fly *Erioptera gemina* [21] or the common house mosquito *Culex pipiens quinquefasciatus* [22, 23].

However, little is known about the impact of the way markers are used on species evolution. Indeed, as both males and females of certain species seem to be able to form swarms over markers, it suggests that these markers could be an important cue in sexual encounters. Moreover, as species seem to use different markers, these probably play an important role in spatial segregation behaviors. Differences in swarming behaviors could be products of past evolutionary traits on which selection is working, especially in closely related species [24].

Mosquitoes of the *Anopheles gambiae* complex, the main malaria vectors in sub-Saharan Africa, are among the insects that mate in swarms [16, 25–32]. Because these species are of medical interest, a particular attention has been paid to their mating biology and ecology. Nowadays, it is known that mating occurs outdoors at sunset in swarms formed by males in which virgin females come to mate [32–36]. Within the complex, *An. coluzzii* and *An. gambiae* (*s.s.*) (formerly the M and S molecular forms of *An. gambiae* (*s.l.*), respectively) are sympatric in west Africa and are considered as two different species [37]. They have been described to have a swarm spatial segregation in which *An. coluzzii* mainly swarms over a ground marker and *An. gambiae* (*s.s*.) over bare ground [29, 32, 38]. In Burkina Faso and Mali, hybrids occur at low frequency (≈ 1%) [32, 39–42] and no evidence for selection against these hybrids was found [42]. There is also no evidence of genetic incompatibilities between parental taxa in experimental crosses, with no obvious loss in the fitness of hybrids in laboratory settings [43–46]. This suggests that reproductive isolation between *An. coluzzii* and *An. gambiae* (*s.s.*) is primarily achieved by distinct pre-mating behaviors [30, 45, 47]. However, the exact nature of these pre-mating barriers between the two sibling species, the mechanisms at the origin of male swarm segregation and the way females are attracted to species-specific swarms are unknown. Such knowledge could contribute in explaining the diversification and evolution within the genus *Anopheles* and could raise new avenues in the development of new control tools.

In this study we investigated the role of visual ground markers in *An. coluzzii* and *An. gambiae* (*s.s.*) swarm segregation and in female attraction under both laboratory and semi-field conditions. As visual markers seem to be a common trait for most swarming insect systems [16–18], we began by predicting that both *An. coluzzii* and *An. gambiae* (*s.s.*) use ground visual markers as landmarks to produce a stationary flight. However, since *An. gambiae* (*s.s.*) was described swarming over bare ground instead of over a marker like *An. coluzzii*, we assumed that males of *An. gambiae* (*s.s.*) use markers at a distance to locate their swarm. Next, since females of other species of mosquito were described flying over similar markers used by males [21–23], we predicted that females of *An. coluzzii* and *An. gambiae* (*s.s.*) also use ground markers as visual landmarks in search for a mate or a mating place in the same way their respective males do. This could lead females to fly continuously at the same locations in the absence of males or at least to repeatedly come and go at these locations. Finally, marker size having an effect on swarm size and swarm characteristics [16–18, 48], we predicted that both *An. coluzzii* and *An. gambiae* (*s.s.*) would increase their swarm size with larger markers. It is however difficult to predict the impact of a larger marker on the swarm shape.

## Methods

### Mosquitoes

For logistic constraints and after preliminary results ensuring that both laboratory-reared and field mosquitoes expressed the same swarming behavior, we chose to use laboratory *An. gambiae* (*s.s.*) (*c*.F10) (hereinafter *An. gambiae*) and field *An. coluzzii* mosquitoes (F1) for indoor experiments and exclusively field mosquitoes for semi-field experiments. The laboratory colony was established in 2015 from wild mosquitoes collected near Bobo-Dioulasso, Burkina Faso (see Additional file 1: Text S1) and F1 mosquitoes were obtained from the same locality. The sex of the mosquitoes was determined early after emergence and males and females were kept in separate 30 × 30 × 30 cm cages to prevent mating before experiments.

### Experimental setups

The laboratory and semi-field experiments were performed in setups described by Niang et al. [49]. Briefly, the laboratory setup (swarming room) is a room without windows measuring 5.10 m in length, 4.70 m wide and 3 m high and equipped with a set of stimuli designed to induce swarming behavior. Among these stimuli: (i) the ambient light is dimmed from full light to full dark in a 30 min time set to simulate sunset and to initiate the swarming behavior; (ii) a bright horizon on one wall allows the swarming behavior to last and to observe mosquitoes. The semi-field setup is made of 12 compartments (10 × 6 × 4.5 m each; L × W × H) with walls made of polyester net with 346 holes/inch^2^ allowing airflow and natural light diffusion (for details see [49]).

### Experimental designs

#### Relative swarm location to the ground marker (laboratory design 1)

**Fig. 1:**
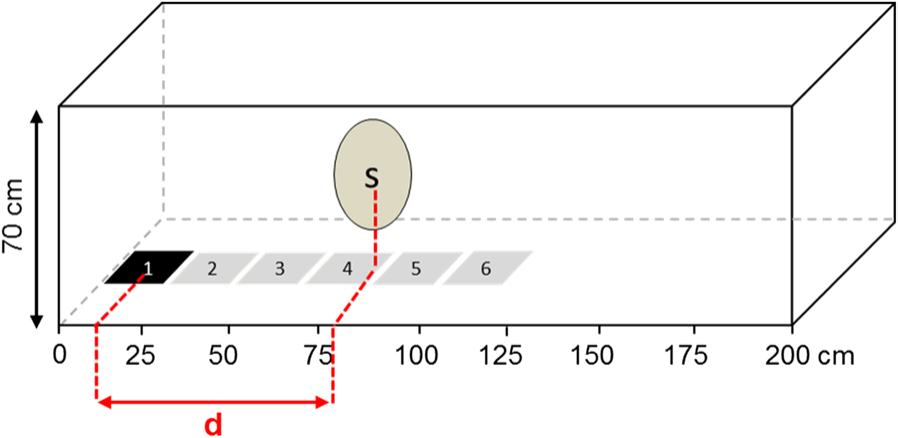
Experimental design for laboratory design 1. The black square with the number 1 represents the 20 × 20 cm black marker and its location for the first and the last measurements (forward/backward movements). Grey squares with the number 2–6 represent the successive locations of the marker for which swarm location was also recorded (forward and backward). Abbreviations: d, the calculated marker-swarm distance; S, the swarm

About 250 four- to six-day-old virgin males of *An. gambiae* or *An. coluzzii* were released into a horizontal cage (200 × 70 × 70 cm; L × W × H) at least 1 h for acclimatisation before the programmed sunset start. The cage frame, painted in white, was covered with white net and placed in the middle of the room. The cage was elevated 10 cm above the ground allowing to place and move a 20 × 20 cm black cloth used as a ground visual marker under the cage. When the programmed sunset started, the marker was randomly placed at one extremity of the cage or the other (Fig. 1). Five minutes after the ceiling lights went off, the locations of the marker center and the swarm nucleus (i.e. the higher density of mosquitoes in the swarm) were recorded. The measurements were based on a graduated adhesive tape stuck along the cage frame. Then, the marker was moved 20 cm further towards the center of the cage. The swarm was slightly disturbed but acquired its new location within seconds. Two minutes later the new locations of both the swarm nucleus and the marker center were recorded. Five successive similar moves were made in the direction of the center of the cage to get 6 marker location records. Then the marker was moved back in 5 moves (= 5 more marker locations recorded) (Fig. 1). These deliberate forward/backward movements allowed to test for potential repulsive and/or attractive effects of the marker on the swarm. Distance between the swarm nucleus and the marker center was calculated and defined as the distance between the marker and the swarm.

#### Swarm characteristics according to marker presence and size (laboratory design 2)

To assess the effect of the marker on the swarmʼs characteristics, *c*.300 four- to six-day-old virgin males of *An. gambiae* or *An. coluzzii* were released into a vertical cage (70 × 70 × 150 cm; L × W × H) as described above. No marker was present. Ten minutes after the ceiling lights went off, presence/absence of swarming mosquitoes was visually detected and recorded. When a swarm was present, 5 characteristics were recorded: (i) minimal and (ii) maximal height, (i.e. the lowest and highest heights at which swarming mosquitoes flew, respectively); (iii) height of the nucleus (i.e. the height at which mosquito density in the swarm was the highest); (iv) width (i.e. the largest horizontal space occupied by the swarm); and (v) swarm size, (i.e. the estimated number of mosquitoes by sight in the swarm at the time of the measurement). In addition, the amplitude, (i.e. vertical space occupied by swarming mosquitoes) was calculated as follows: amplitude = maximal − minimal height. As before, measurements were done thanks to graduated adhesive tape stuck on the cage frame (one vertical for height measurements and another horizontal for width measurements). Although done by sight, the estimations of the number of mosquitoes in the swarms is accurate when performed by experienced people [49]. Once the first recordings in the absence of the marker were made, a 20 × 20 cm or 60 × 60 cm marker (20 cm and 60 cm, hereafter) was placed either into the cage for *An. coluzzii* or at a distance of 20 cm outside the cage for *An. gambiae*. The choice of these marker locations was based on the results obtained from the first experiment and allowed to obtain swarm in the cage center according to the species. Five minutes after introducing the marker, swarm characteristics were recorded again. Then, the marker was removed and a second round of measurements in the absence, and then, in the presence of the same marker were done. Consequently, each parameter was measured twice during a test. This alternation of presence/absence of the marker allowed to reduce both the sequential introduction effect (i.e. measurements first made with or without swarming mosquitoes) and a swarm temporal size effect. Indeed, the number of mosquitoes in the swarm is not constant over time and tends to increase before reaching a maximum after *c*.8 min, becoming consistent during 20 min and then decreasing at the end of the swarming window time (SBP, personal observation). Consequently, the four sequential measurements were done within the swarm peak window time. For each parameter measured, data of the two sessions of presence/absence of markers were combined and the means were used in statistical analysis.

#### Female swarming behavior

The two experimental designs were repeated with two- to four-day-old virgin females of the two species as described above for males. However, the method was slightly modified for *An. gambiae* females. Indeed, preliminary tests showed that swarming *An. gambiae* females did not stay at the swarming location for as long as the males, but came at the swarm location and did few loops before leaving (see the Results section). Consequently, it was not possible to record some data by eye such as swarm maximal and minimal heights and width. Thus, we adapted our observational method to gather some data about this specific swarm-like behavior. Here, three parameters were recorded: (i) the number of females doing more than 2 consecutive loops (i.e. flying consistently in the center of the cage = one event); (ii) their flight height; and (iii) the number of events during the 5 min of observation in presence or absence of a marker (i.e. swarming frequency). Considering females which did more than two loops allowed to discard females which did a simple U-turn in the middle of the cage. This continuous recording method was adopted for *An. gambiae* females only.

#### Effect of insemination on female swarming behavior (in the laboratory)

The purpose of this experiment was to determine if swarming females are searching for a mate (in that case, only virgin females would swarm) or if they swarm anyway (both inseminated and virgin females would swarm in this case). To test the effect of the mating physiological status on the motivation of females to produce swarm-like behaviors, we repeated experimental design 2 with inseminated females.

After emergence, females were allowed to mate with males for 3 consecutive nights in 30 × 30 × 30 cm cages and provided with a 5% glucose solution. Each cage contained about 500 mosquitoes with a sex ratio of two males for one female. Following the three mating nights, 50 females were collected in each cage and the insemination rate was assessed through the dissection of the spermathecae under a microscope (× 400 Leica DM750, Leica, Wetzlar, Germany). About 300 “inseminated females” were then used for the experiment using the 20 cm marker only, as in design 2. After the swarm’s characteristics were recorded, swarming females were collected with a net and their insemination status was assessed and compared to the whole cage insemination rate. Because *An. gambiae* females exhibited an unstable swarm-like behavior, the collection of swarming females to check for their insemination status was conducted with *An. coluzzii* females only.

Ten replicates per sex and species combination were performed in the first experimental design and 12 replicates per marker size, sex and species combination were performed in the second experimental design. All observations were performed by only one observer to avoid observer bias. The observer was located at about 1.5 m from the cage and the cage was located between the observer and the bright horizon which allowed good conditions (contrast) for the observation of flying mosquitoes without disturbing swarming behavior [49] (no obvious alteration of the swarm characteristics was observed when the observer moved his position in the room). Repeatability of measurements by the observer was tested and the mean standard error was < 2 cm which was below the lowest significant difference found in our analyses.

#### Swarm relative location to the marker and swarm characteristics in semi-field setups

To validate our results and exclude any artefact due to cage effects in the laboratory experiments, the same questions developed in both designs 1 and 2 were investigated in a single experiment in the semi-field setup on both males and females and separately for the two species. Two different sizes of ground visual markers (100 × 100 cm and 60 × 60 cm black cloths, large and small hereafter, respectively) were used to evaluate the effect of marker size on swarm characteristics and the 60 × 60 cm marker was used to evaluate the relative location of swarm. About 300 four- to five-day-old males or 300 three- to four-day-old virgin females were transported from the insectarium to the semi-field setup in a cage (20 × 20 × 20 cm) *c*.2 h before sunset. Mosquitoes were released from the cage into a compartment of the semi-field setup 30 min before sunset and the large marker was placed into the compartment. Observations and swarm characteristic measurements started 8 min after the first males started to swarm. The same characteristics used in experimental design 2 were measured for the swarm as well as the distance separating it from the marker. Then, the large marker was replaced by the small one at the same location and swarm characteristics were recorded again 5 min later (= effects of marker size). Secondly, the small marker was moved twice about 210 cm and the distance between the swarm and the marker was measured each time 5 min after the marker was moved (= swarm relative location). Each mosquito batch was used only once. For each species, 10 and 15 replicates were performed for males and females, respectively. As before, accurate measurements were made possible thanks to graduated adhesive tape stuck on a wall (Additional file 1: Figure S1). Experiments were carried out simultaneously in two adjacent compartments. The measurements were done by two observers (an observer per compartment). The observer/compartment combination was fixed between replicates but species and sexes were randomly assigned to one compartment or the other each day. Swarms of *An. gambiae* females were less stable than those of *An. coluzzii*, but swarm characteristics were measurable.

### Data analysis

All analyses were performed using R (version 3.4.0). We analyzed the effects of marker location, marker size, mosquito species, sex, insemination status, number of released and swarming mosquitoes and some interactions on (i) marker-swarm distance (laboratory design 1) and (ii) swam characteristics (minimal, nucleus and maximal heights, swarm width and amplitude, number of swarming mosquitoes and swarming frequency) (laboratory design 2) using generalized linear mixed models (GLMM, lme4 package) with the appropriate distribution family.

Data from laboratory and semi-field experiments were analyzed separately. In addition, since the protocols used in the laboratory study for marker size effects (laboratory design 2) were different for *An. coluzzii* and *An. gambiae* (i.e. location of the marker inside or outside the cage, respectively), different species subsets were used and analyzed separately. Similarly, the protocol used to observe *An. gambiae* females in the laboratory experiments was different than the one for males, consequently, they were also analyzed separately. Additional file 1 provides information on full statistical models and model selection.

## Results

A total of 160 swarms were observed in the laboratory (68 and 92 for males and females, respectively) and 50 swarms in semi-field conditions (20 and 30 for males and females, respectively). What we call swarm or swarming behavior was consistent with the definition provided by Downes [16], i.e. a constant up-and-down or to-and-from flight at a particular station and within constant boundaries of a single individual or several individuals. In the laboratory, about 5 min before the ceiling lights went off, some males had erect antennae and randomly flew in the whole cage volume. Immediately after the ceiling lights went off in the laboratory or a few minutes after sunset in semi-field conditions, swarms were initiated by 2–3 males. The number of mosquitoes in swarms gradually increased and swarms reached their maximal size about 5 min later. The number of swarming mosquitoes gradually decreased after 25–30 min. Non-swarming mosquitoes were observed resting or flying and bouncing on the walls. We also observed that some females exhibited a swarm-like behavior. This behavior was very different than that of females activated by the light conditions and flying randomly and bouncing on the net. Moreover, since we observed the same behavior in semi-field setups, we can exclude an effect of cage size. Nevertheless, while *An. coluzzii* female swarms were very similar to male swarms, those formed by *An. gambiae* females were less stable with one or a few females coming at the swarming location, doing a few loops and leaving.

### Swarm relative location to the ground marker (laboratory and semi-field setups)

**Fig. 2:**
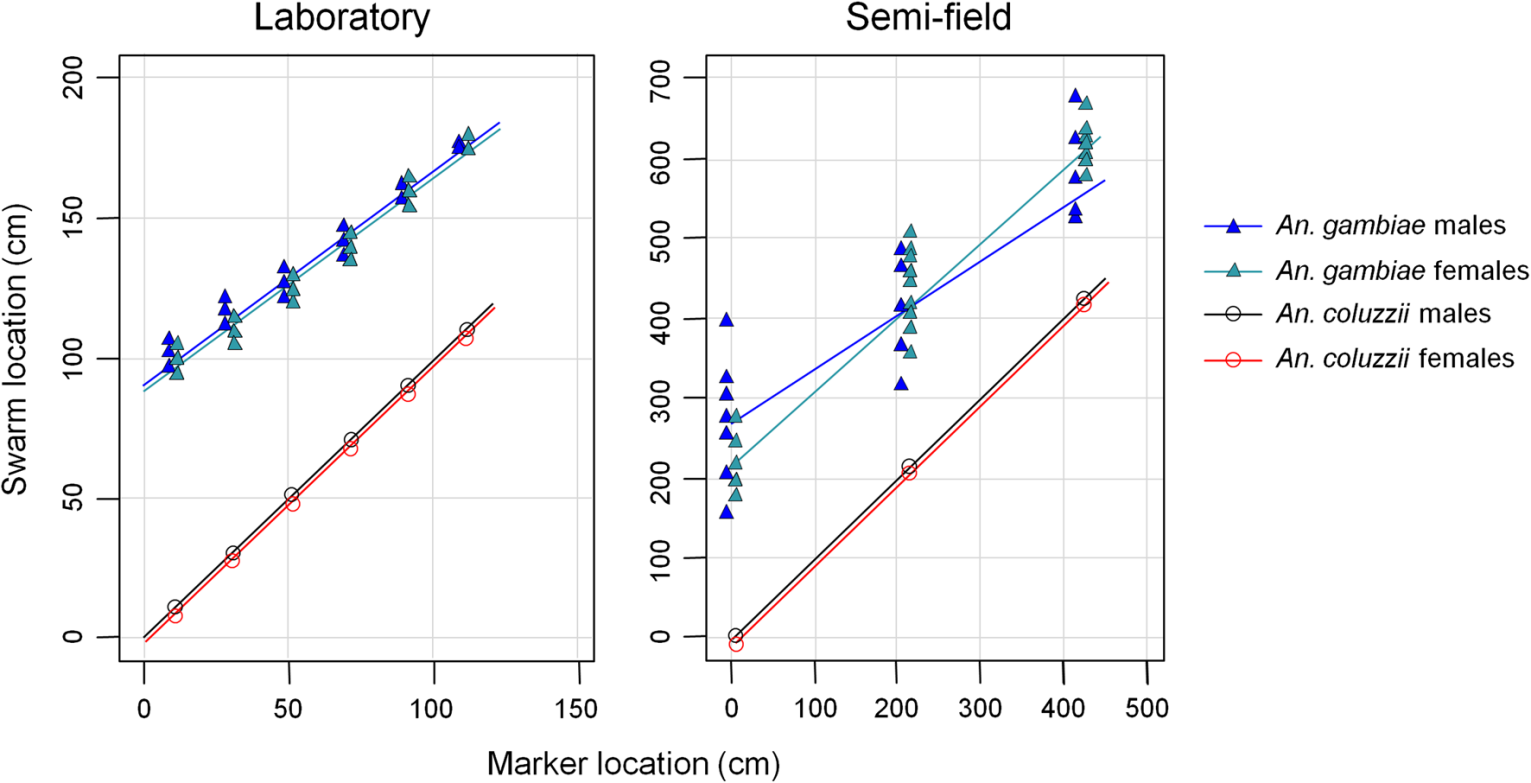
Relative location of both male and female swarms of *Anopheles gambiae* (*s.s*.) and *Anopheles coluzzii* to the marker in both laboratory and semi-field setups. Due to some perfect overlaps of female and male data, some points and lines have been voluntarily slightly shifted for a better visualization

Relative swarm location to the marker was species-specific (species: $\chi_{1}^{2}$ = 21,881, *P* < 0.001 and $\chi_{1}^{2}$ = 0.78, *P* < 0.001 in laboratory and semi-field setups, respectively) with *An. coluzzii* swarming right over the marker and *An. gambiae* swarming at a distance from the marker (76.4 ± 0.6 cm and 206 ± 6 cm in laboratory and semi-field setups, respectively, Fig. 2). This difference between species was consistent regardless of the marker location (maker location: $\chi_{1}^{2}$ = 0, *P* = 1 in both laboratory and semi-field setups). Both conspecific males and females swarmed at the same location (sex: $\chi_{1}^{2}$ = 2.53, *P* = 0.11 and $\chi_{1}^{2}$ = 0, *P* = 1 in laboratory and semi-field setups, respectively, Fig. 2). There was a marker location × species interaction ($\chi_{1}^{2}$ = 567.5, *P* < 0.001 and $\chi_{1}^{2}$ = 6.42, *P* = 0.04 in laboratory and semi-field setups, respectively) with a decrease in marker-swarm distance in *An. gambiae* when the marker was moved toward the cage or compartment center whereas *An. coluzzii* swarms followed the marker and kept their location right above it. No significant interaction was found between marker location and sex ($\chi_{1}^{2}$ = 0.32, *P* = 0.57 and $\chi_{1}^{2}$ = 0, *P* = 1 in laboratory and semi-field conditions, respectively). A significant interaction was found between species and sex in the semi-field setup ($\chi_{1}^{2}$ = 13.15, *P* < 0.001) but not in the laboratory ($\chi_{1}^{2}$ = 2.83, *P*= 0.09) (Fig. 2).

In the semi-field setup, marker size, mosquito sex and the number of swarming mosquitoes significantly influenced the marker-swarm distance in *An. gambiae* ($\chi_{2}^{2 }$ = 364, *P* < 0.001; $\chi_{1}^{2}$ = 15.9, *P* < 0.001; and $\chi_{1}^{2}$ = 5.7, *P* = 0.017, respectively). The distance between the marker and the mosquitoes was greater with the large marker than with the small one (281 ± 13 cm *vs* 230 ± 11 cm, respectively). Female swarms were closer to the marker than male swarms (229 ± 6 cm *vs* 302 ± 17 cm, respectively) and the swarm was closer to the marker when the number of swarming mosquitoes was small. The interaction between marker size and sex was found to be marginally significant ($\chi_{1}^{2}$= 3.9, *P* = 0.04).

### Swarm characteristics according to marker presence and size (laboratory and semi-field setups)

#### *Anopheles coluzzii* males and females

**Fig. 3:**
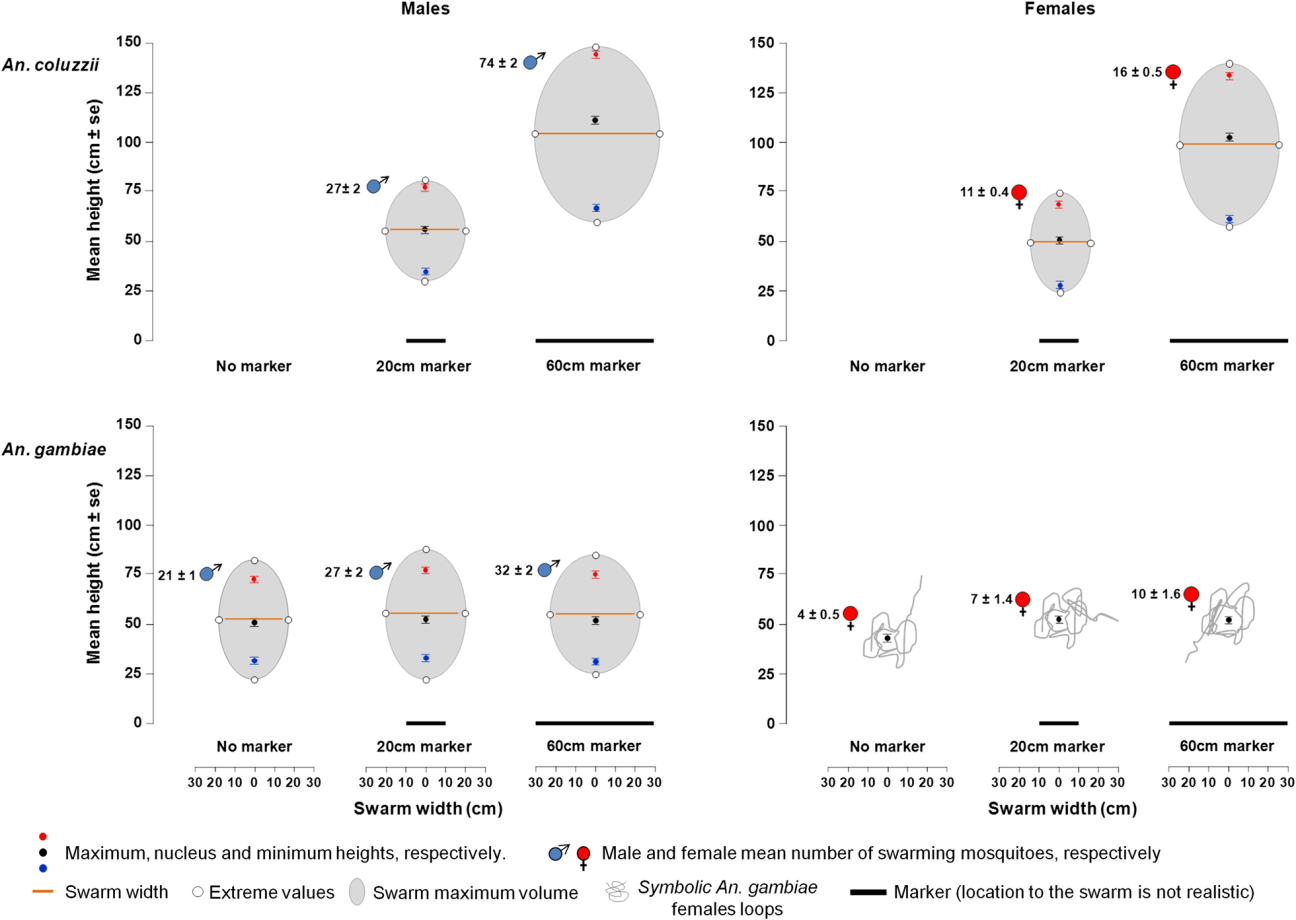
Swarm characteristics in both males and females of *Anopheles gambiae* (*s.s*.) and *Anopheles coluzzii* according to the presence/absence and the size of a ground marker in the laboratory setup. The mean heights and mean mosquito numbers were provided with their standard error. *Anopheles coluzzii* was found only to swarm over the ground marker

In the laboratory, both *An. coluzzii* males and females swarmed only in the presence of a marker. Marker size significantly affected all swarm characteristics, the large marker recruited more mosquitoes and these flew higher and produced swarms which were horizontally and vertically more stretched than with the small marker (Fig. 3, Table 1). No difference was detected between sexes in their minimal, maximal or nucleus heights nor in swarm amplitude according to the marker size (Fig. 3, Table 1). However, male swarms were significantly wider than female swarms and swarming males were more numerous than females (Fig. 3, Table 1). All the swarm dimensions, aside from nucleus height, were positively affected by the number of swarming mosquitoes (Table 1). However, the number of swarming mosquitoes was negatively affected by the number of mosquitoes in the cage ($\chi_{1}^{2}$ = 4.37, *P* = 0.036). There were significant interactions between the number of swarming mosquitoes and the sex regarding maximal height, and both swarms’ width and amplitude. These measurements increased for both sexes but more rapidly in females than in males and required a lower number of recruited swarming females (Additional file 1: Table S1). Moreover, there was a significant interaction between marker size and the sex on the number of swarming mosquitoes, with both males and females being more numerous over the large marker compared to the small marker, but this increase was larger in males than in females (Additional file 1: Table S1). The interaction between marker size and the number of swarming mosquitoes on the minimal swarm height was marginally significant with the swarm minimal height increasing more rapidly with the small marker than with the large one depending on the recruited mosquitoes (Additional file 1: Table S1). All other interactions were not significant (Additional file 1: Table S1).

**Table 1:** Effects of marker size, mosquito sex, number of swarming mosquitoes and number of released mosquitoes on swarm characteristics in *An. coluzzii* in laboratory setups (design 2)

	Marker size^a^					Mosquito sex					No. of swarming mosquitoes		
	20 cm	60 cm	*χ*^2^	*df*	*P*-value	Males	Females	*χ*^2^	*df*	*P*-value	*χ*^2^	*df*	*P*-value
Swarm dimensions (± SE) (cm)													
Maximal	73.0 ± 1.1	139.0 ± 1.3	168.90	1	**< 0.001**	110.9 ± 7.0	101.0 ± 6.8	0.48	1	0.484	11.87	1	**< 0.001**
Nucleus	53.2 ± 0.9	106.8 ± 1.2	134.30	1	**< 0.001**	84.0 ± 5.8	76.0 ± 5.5	0.0003	1	0.980	2.09	1	0.147
Minimal	31.1 ± 0.9	64.3 ± 0.9	360.80	1	**< 0.001**	50.7 ± 3.5	44.7 ± 3.5	2.99	1	0.083	35.89	1	**< 0.001**
Amplitude	41.9 ± 0.9	74.7 ± 1.0	74.06	1	**< 0.001**	60.2 ± 3.7	56.4 ± 3.4	2.59	1	0.107	4.97	1	**0.025**
Width	32.0 ± 0.8	52.1 ± 1.8	51.38	1	**< 0.001**	47.4 ± 2.7	36.7 ± 1.7	6.01	1	**0.014**	9.07	1	**0.002**
Swarm size^b^ (± SE)													
No. of swarming mosquitoes	19.2 ± 1.9	44.9 ± 6.1	10.65	1	**0.001**	50.5 ± 5.1	13.7 ± 0.6	76.73	1	**< 0.001**	na	na	na

^a^*An. coluzzii* mosquitoes swarmed only in presence of a marker

^b^Swarm size expressed as the number of swarming mosquitoes

*Abbreviations*: SE, standard error; na, not included in the analyses

*Note*: Significant *P*-values are in bold

**Fig. 4:**
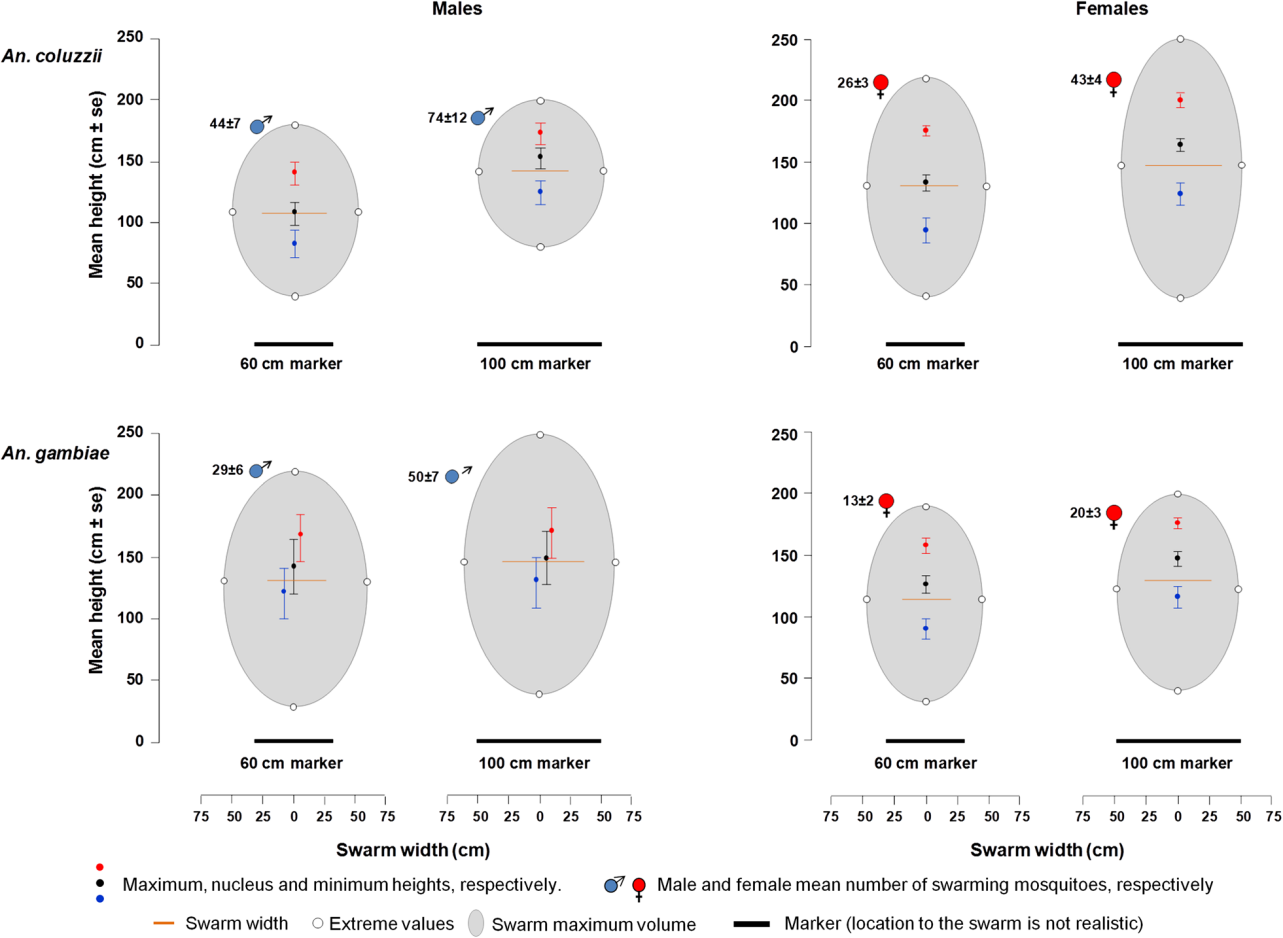
Swarm characteristics in both males and females of *Anopheles gambiae* (*s.s.*) and *Anopheles coluzzii* according to the size of a ground marker in the semi-field setup. The mean heights and mean mosquito numbers were provided with their standard error. *Anopheles coluzzii* was found only to swarm over the ground marker

Similar results were obtained in semi-field experiments. The large marker recruited more *An. coluzzii* males which flew higher and in bigger swarms than over the small marker. However, unlike in the laboratory setup, the number of mosquitoes in the swarm had no effect on the swarm’s characteristics (Fig. 4, Table 2, Additional file 1: Table S2).

**Table 2:** Effects of marker size, mosquito sex, number of swarming mosquitoes and number of released mosquitoes on swarm characteristics in *An. coluzzii* and *An. gambiae* in semi-field setups

	Marker size					Species					Sex					No. of swarming mosquitoes		
	60 cm	100 cm	*χ*^2^	*df*	*P*-value	*An. coluzzii*	*An. gambiae*	*χ*^2^	*df*	*P*-value	Female	Male	*χ*^2^	*df*	*P*-value	*χ*^2^	*df*	*P*-value
Swarm dimension (± SE) (cm)																		
Maximal	161.5 ± 4.8	181.6 ± 4.9	32.19	1	**< 0.001**	176.0 ± 4.2	167.2 ± 5.7	2.20	1	0.14	177.5 ± 3.1	161.8 ± 7.8	6.87	1	**0.009**	0.0047	1	0.95
Nucleus	127.4 ± 5.5	153.6 ± 4.9	70.92	1	**< 0.001**	140.9 ± 4.5	140.4 ± 6.4	1.92	1	0.17	142.4 ± 3.5	137.7 ± 8.6	0.07	1	0.79	0.22	1	0.64
Minimal	95.9 ± 6.1	122.6 ± 5.7	31.90	1	**< 0.001**	107.3 ± 5.6	111.5 ± 6.8	0.14	1	0.71	106.4 ± 4.8	114.3 ± 8.4	0.38	1	0.54	0.013	1	0.91
Amplitude	65.6 ± 44.9	58.6 ± 3.8	2.81	1	0.09	68.3 ± 4.3	57.7 ± 3.9	3.40	1	0.06	71.1 ± 3.3	46.9 ± 4.5	11.46	1	**< 0.001**	0.19	1	0.67
Width	45.3 ± 3.3	55.2 ± 3.4	0.018	1	0.89	51.3 ± 3.2	49.3 ± 3.6	0.35	1	0.55	49.5 ± 3.2	51.5 ± 3.6	1.29	1	0.26	5.3	1	**0.021**
Swarm size^a^ (± SE)																		
No. of swarming mosquitoes	26.1 ± 2.6	42.8 ± 3.9	228.75	1	**< 0.001**	43.5 ± 3.7	25.3 ± 2.7	19.06	1	**< 0.001**	25.3 ± 2.1	49.4 ± 4.8	22.33	1	**< 0.001**	na	na	na

^a^Swarm size expressed as the number of swarming mosquitoes

*Abbreviations*: SE, standard error; na, not included in the analyses

*Note*: Significant *P*-values are in bold

#### *Anopheles gambiae* males and females

In the laboratory setup, both *An. gambiae* males and females were able to form swarms in both the presence and “absence” of a marker. In males, both the swarm’s width and the number of swarming mosquitoes increased when a marker was present and when the marker size increased (Fig. 3, Table 3). However, neither the minimal, maximal or nucleus heights, nor the swarm’s amplitude were affected by the marker’s presence/absence or by its size (Fig. 3, Table 3). The increasing number of swarming males formed a significantly inflated swarm with an increase of both width and maximal height, a decreased minimal height and consequently a larger amplitude (Table 3). However, the nucleus height was not affected by the number of swarming males (Table 3). The number of swarming males was positively correlated with the number of mosquitoes in the cage (Table 3). No significant interaction was found between the marker and the number of swarming males on the swarm’s characteristics (Additional file 1: Table S3).

**Table 3:** Effects of marker size, number of swarming mosquitoes and number of released mosquitoes on swarm characteristics in *An. gambiae* in laboratory setups (design 2)

		Marker size						Number of swarming mosquitoes			Number of released mosquitoes		
		Ø	20 cm	60 cm	*χ*^2^	*df*	*P*-value	*χ*^2^	*df*	*P*-value	*χ*^2^	*df*	*P*-value
Males	Swarm dimensions (± SE) (cm)												
	Maximal	72.1 ± 1.1	76.9 ± 1.6	75.0 ± 1.3	4.97	2	0.083	9.72	1	**0.001**	na	na	na
	Nucleus	50.9 ± 1.0	52.5 ± 1.4	51.5 ± 1.2	1.56	2	0.462	3.29	1	0.072	na	na	na
	Minimal	31.8 ± 1.1	33.3 ± 1.5	31.5 ± 1.4	0.88	2	0.641	10.84	1	**< 0.001**	na	na	na
	Amplitude	40.3 ± 1.6	43.5 ± 2.4	43.5 ± 2.0	3.81	2	0.148	23.09	1	**< 0.001**	na	na	na
	Width	29.1 ± 0.9^b^	34.4 ± 1.6^c^	39.8 ± 1.2^d^	25.87	2	**< 0.001**	11.95	1	**< 0.001**	na	na	na
	Swarm size^a^ (± SE)												
	No. of swarming mosquitoes	21.5 ± 1.3^b^	27.1 ± 2.2^c^	32.3 ± 2.3^d^	27.14	2	**< 0.001**	na	na	na	21.19	1	**< 0.001**
Females	Swarm dimensions (± SE) (cm)												
	Height	43.4 ± 1.1^b^	52.7 ± 1.4^c^	51.8 ± 1.1^c^	91.04	2	**< 0.001**	5.56	1	**0.018**	na	na	na
	Swarm size (± SE)												
	No. of swarming mosquitoes	3.7 ± 0.5 ^b^	7.0 ± 1.4^c^	9.6 ± 1.6^c^	44.12	2	**< 0.001**	na	na	na	0.02	1	0.871
	Swarming frequency (no./5min ± SE)												
	Frequency	9.3 ± 0.3^b^	15.0 ± 0.3^c^	15.5 ± 0.3^c^	105.83	2	**< 0.001**	na	na	na	0.22	1	0.632

^a^Swarm size expressed as the number of swarming mosquitoes

*Abbreviations*: Ø, absence of marker; SE, standard error; na, not included in the analyses

*Notes*: Significant *P*-values are in bold. Different superscript letters indicate significant differences between marker sizes (*P <* 0.05)

Results obtained in the semi-field experiment with *An. gambiae* males were similar to those obtained in the laboratory setup. The large marker recruited more males which produced a wider swarm than with the small marker. Although females have unstable swarms, the results were also similar to those of the males. However, none of the maximal, nucleus or minimal heights were affected by the marker size. Unlike the laboratory experiments, the number of swarming mosquitoes did not affect any of the swarm’s characteristics in both males and females of *An. gambiae* (Fig. 4, Table 2, Additional file 1: Table S2).

In the laboratory setup, the swarming behavior of *An. gambiae* females was different from the males, few females came at the swarming spot at the same time and they only performed a few loops before leaving. Females flew higher, came more frequently at the swarming spot and in a greater number in the presence of a marker. However, the marker size did not have any effect on these parameters (Fig. 3, Table 3). The swarm height significantly increased with the number of swarming females (Table 3). The number of females in the cage did not affect the number of swarming females nor the loop frequency (Table 3). None of the interactions tested was found to be significant (Additional file 1: Table S3).

### Effect of insemination on female swarming behavior (laboratory setup only)

The effect of the insemination status on female swarming behavior was tested in both species but only in the laboratory setup. For the record, we compared female swarming behaviors using two sets of females: a set composed of virgin females only; and a set of females which had time to be inseminated by males. Only 22% of females collected in the swarms were inseminated females (3 over 27 swarming females collected) which was significantly lower than the insemination rate of the whole female population released in the cage (79%) ($\chi_{1}^{2}$ = 51, *P* < 0.001).

In both *An. coluzzii* and *An. gambiae*, the number of swarming females was negatively affected by the insemination status (virgin *vs* “inseminated”: 11.22 ± 0.36 *vs* 5.08 ± 0.37 swarming *An. coluzzii* females, $\chi_{1}^{2}$ = 22.33, *P* < 0.001; and 5.16 ± 0.85 *vs* 0.54 ± 0.08 swarming *An. gambiae* (*s.s.*) females, $\chi_{1}^{2}$ = 19.49, *P* < 0.001) independently from the number of mosquitoes in the cage ($\chi_{1}^{2}$ = 0.37, *P* = 0.54 in *An. coluzzii* and $\chi_{1}^{2}$ = 0.57, *P* = 0.45 in *An. gambiae*). No significant interaction was found between the insemination status and the number of mosquitoes in the cage ($\chi_{1}^{2}$ = 0.006, *P* = 0.94 in *An. coluzzii* and $\chi_{1}^{2}$ = 0.24, *P* = 0.62 in *An. gambiae*).

In *An. gambiae*, the frequency at which swarming females did loops at the swarming spot was also negatively affected by the insemination status (virgin *vs* “inseminated”: 11.97 ± 0.65 *vs* 0.72 ± 0.13 times/5 min, respectively, $\chi_{1}^{2}$ = 59.35, *P* < 0.001) but not by the number of females in the cage ($\chi_{1}^{2}$ = 0.53, *P* = 0.46). No significant interaction was found between the insemination status and the number of females in the cage ($\chi_{1}^{2}$ = 0.1, *P* = 0.75).

## Discussion

Aggregation above a visual marker is a common trait to most swarming insect systems [16–18]. However, in the *An. gambiae* complex, the effect of these markers on male swarm characteristics and their role in female attraction remains unknown. Our main findings corroborate field observations reporting that *An. coluzzii* mosquitoes swarm over visual ground markers and *An. gambiae* mosquitoes swarm over bare ground [29, 32, 38] but in the present study we report new data on *An. gambiae* mosquitoes which use visual markers at a distance to locate their swarms. Moreover, we show that females also display a swarm-like behavior which was dependent of their insemination status with inseminated females being less likely to participate in female “swarms”. These new behaviors were very similar in both laboratory and semi-field setups.

More precisely, as expected, we observed a species-specific location of *An. coluzzii* and *An. gambiae* male swarms in relation to the marker. This result on *An. gambiae* is different from what was initially believed as this species is supposed to swarm over bare ground independently of the presence of a visual marker [29, 32, 38]. The same pattern was described in other closely related species such as *Culicoides nubeculosus* which swarms above a marker while *Culicoides grisescens* swarms near conspicuous erected objects [18], or in the case of mayfly *Leptophlebia marginata,* where different sympatric populations genetically distinct swarm either over or beside trees [50]. Downes [18] hypothesized that physiologically this distinct behavior could be explained by different visual abilities, species swarming over landmarks may watch the marker with the ventrally directed ommatidia, while those swarming next to the marker are more likely to use the lateral ommatidia, but this hypothesis has to be tested in *Anopheles* species.

Deliberately moving the ground markers induced an equivalent movement of swarms in the two species with *An. coluzzii* keeping its location above the marker and *An. gambiae* its distance to the marker. Other swarming insects were observed tracking marker movements as is the case with the mosquito *Culex fatigans* [5] and the chironomid *Spaniotoma minima* [51]. These observations provide evidences that ground visual markers are used not only to form the swarms but also as landmarks to stabilize the swarm’s location and, as a byproduct, to keep the mosquitoes gathered in the swarm when the marker is moved or, in more natural conditions, when the swarm is disturbed by predators or wind. However, in *An. gambiae*, the distance between the swarm and the marker was slightly reduced when the swarm was moved closer to the wall of the flight arena. These results can be explained by an edge effect, as the marker was moved the swarm was brought closer to the wall which had a repulsive effect on the swarm. When the distance to the wall was too short, the swarm was disrupted and reformed on the other side of the marker (SBP, personal observation). Moreover, the swarm-marker distance was 2.7 times longer in the semi-field setup than in the laboratory experiment (76 *vs* 206 cm, respectively). As before, this could be due to a wall effect, mosquitoes in the semi-field setup being less constrained than in the laboratory cage, and/or an effect of the marker size which was larger in the semi-field setup than in the laboratory (60 *vs* 20 cm each side, respectively). Consequently, one can expect that in natural conditions (no walls) the distance between markers and *An. gambiae* swarms could be larger which could explain why the link between swarm and marker was not established. Moreover, in natural conditions, additional aspects could be involved in swarm location which would prevent *An. gambiae* from forming several swarms all around a marker. Swarms of *An. coluzzii* and *An. gambiae* are also rarely observed as close to each other as suggested by our results. This could be avoided by the presence of more numerous and diversified markers among which some would be more attractive than others according to the species.

Marker size had strong effects on swarm characteristics. In the two species, large markers recruited more mosquitoes than small ones, as it has already been observed in *Culicoides brevitarsis* and *C. nubeculosus*, in the midge *Anarete* sp. near *felti*, in species of blood-sucking snipe flies (Rhagionidae), and in the mosquitoes *Anopheles maculipennis* and *Culex pipiens pallens* [17, 18, 52–54]. Inclusion of additional mosquitoes in the swarm can induce either an increase in mosquito density for a given swarm volume or lead to an inflation of the swarm with the same mosquito density (and all intermediate possibilities). Results obtained in the field with a video tracking system by Manoukis et al. [55, 56] support the intermediate option, both the mosquito density and the swarm size were increased with the inclusion of an additional number of mosquitoes. Our observation method does not allow us to gather information on mosquito density. However, in both laboratory and semi-field setups, we observed that in *An. coluzzii*, the largest markers induced an increase in the swarm size in all directions (horizontal and vertical), while in *An. gambiae*, swarm size increased only in a horizontal dimension (larger swarms). Swarm inflation may either indicate that each male needs a given airspace to produce a stereotyped swarming flight and an increase of the maker size will increase the size of the exploitable “territory”, or that each male explores a larger volume in the swarming area, in which it is susceptible to find a female. In the first case, males would not change their swarming loop amplitude while in the second, loops would be larger. Unfortunately, it is impossible to answer this question by using naked-eye observations. Nevertheless, data on other swarming insects have shown that markers cannot “absorb” an infinite number of individuals in the swarm, additional individuals prefer to form a new swarm over another marker [16, 18, 48, 55, 57] which suggests that each male needs a given airspace over the marker. Concomitantly, increased marker size induced a swarm flying at a larger distance from the marker, higher in *An. coluzzii* and farther in *An. gambiae*. Downes [16, 18] and Sullivan [20] hypothesized that keeping a stationary flight would require a constant view of the marker by using a constant vision angle. Therefore, when the marker gets bigger, increasing the distance to the marker would allow the mosquitoes to visually keep the marker at approximately the same size.

Females exhibited a swarm-like behavior at the same location of their conspecific males and reacted to the marker’s movements. The way they reacted to marker size was also similar to their conspecific males. The fact that this swarm-like behavior was almost exclusively produced by non-inseminated females provides evidences that it was a behavior related to mate-seeking. The small proportion of inseminated *An. coluzzii* females found among the “swarming” females (3/27) could be a bias due to our capture method which did not allow to reliably collect only “swarming” females above the marker, although it is not excluded that some inseminated females of the *An. gambiae* complex may be encountered in natural male swarms [40]. Our results provide the experimental evidence that the virgin females of both *An. gambiae* (*s.s.*) and *An. coluzzii* use ground markers to join conspecific male swarm sites. However, female “swarms” observed in our experimental setups could be due to the absence of males [21–23]. Indeed, natural female swarms of *An. gambiae* (*s.l.*) have never been reported. This is probably because females usually arrive at the swarming spot when a male swarm is already present and thus are quickly inseminated once they enter male swarms [25, 36, 58]. We can hypothesize that in the absence of males in the field, females would have the same behavior than in the laboratory and semi-field setups, they would first come at the swarming site attracted by a marker, and then, instead of being inseminated within a few seconds, females would have a swarm-like flight over or next to the marker searching for a mate and probably leave the swarming spot if no male was encountered.

Sullivan [20] suggested that the height at which males swarm reflect the height at which females approach the swarm, and conversely, we can hypothesize that females “swarm” at the height they expect to find males. However, the height at which swarms occur in the two sexes could be the result of the marker size only, both males and females being attracted by the marker specificity (environmental cue attraction) instead of swarm characteristics (conspecific cue attraction) [4]. To this extent, a difference in swarm height or marker exploitation between closely related species could limit the contact of species in sympatric areas [27] and promote species divergence.

Female “swarms” have already been observed in laboratory settings. In *Cx. pipiens quinquefasciatus*, females reacted to the same visual cues as males [22, 23] suggesting that it could be a common trait in swarming species. In the cranefly *E. gemina*, which is easier to spot individually thanks to its large size, Savolainen & Syrjamaki [21] reported that in the field, when a female arrived over a marker unused by males, females behaved like males reinforcing the idea that both males and females use the same visual cues to find aggregation sites and that the two sexes are capable of swarming behavior.

Despite the fact that our results were obtained by naked-eye observations and with finite mosquito populations, differences recorded in swarm characteristics and in reaction to marker size between *An. coluzzii* and *An. gambiae* were consistent in our two experimental setups indicating that these setups are highly valuable for mosquito swarm studies. Nevertheless, some of these observations are preliminary and would benefit from a 3D video tracking system to be confirmed. Such a device would allow to obtain more accurate observations of flight trajectories and mosquito interactions in the swarm. Some points also need clarifications. Indeed, *An. gambiae* was able to form swarms even in the absence of a marker. However, we cannot be affirmative that the black horizon all around the laboratory or the cage frame were not used as a marker in the absence of a closer or more conspicuous marker, respectively. Similarly, both the cage and the compartment walls were also a constraint in the study of the marker-swarm distance, this provides evidence that mosquitoes were able to detect them and adjust their swarm location. Nevertheless, the observed behaviors are definitively swarming behaviors and not an artifact of the experimental setups: first, the swarming behaviors occurred in both the laboratory and semi-field setups with the same characteristics, the second one being more natural than the first one; secondly, swarm characteristics are species specific and are the same for both conspecific males and females; thirdly, the swarm-like behavior is more frequent in virgin females than in inseminated females; and finally, couples were observed in both laboratory and semi-field setups in some occasions in which males and females were released together (SBP and OR, personal observation).

Speciation within the *An. gambiae* complex could be partially driven by this swarming behavior. Since the two sexes use the same visual cues to locate the swarm and the marker size has the same impact on both male and female swarm characteristics, any single significant change in one sex or in one population may compromise mating and promote divergence. Moreover, our results show that swarm segregation between *An. coluzzii* and *An. gambiae* is related to the use of the same type of markers but in a different way. Consequently, speciation between these two species could be the result of the emergence of a population increasing its distance to the marker which would lead to two distinct species using similar swarming markers. In addition, such difference in swarm location was also observed on several occasions in which males of both *An. coluzzii* and *An. gambiae* were released in the horizontal cage in the laboratory and in the semi-field setup. Two swarms were observed at the expected locations highlighting that this difference is consistent with mixed populations (SBP and OR, personal observation). Actually, the use of different swarming sites between closely related species swarming at the same time has already been reported for ten *Culicoides* and nine *Aedes* species for which no mixed swarm was observed [59]. Consequently, swarming behaviors can be recognized as a mechanism of premating isolation. Moreover, individuals that produce divergent swarming behaviors have generally diverged morphologically and/or genetically [4, 50, 60]. Nevertheless, very few data have been gathered on the evolutionary forces that generate divergence and the mechanisms that maintain genetic isolation in the *An. gambiae* complex [30]. Uncovering the behavioral, ecological and genetic mechanisms involved in their speciation may help to understand how biological diversity is generated and to improve vector control strategies.

## Conclusions

Our study provides experimental evidence that both *An. coluzzii* and *An. gambiae* males use ground visual markers to form and locate their swarm at species specific locations, *An. coluzzii* over and *An. gambiae* next to the marker. Moreover, a large marker recruited more mosquitoes in the two species but had species specific effects on swarm characteristics; with *An. coluzzii* swarm size increased both vertically and horizontally, while *An. gambiae* swarm size only increased horizontally and flew higher. In addition, our results show that virgin females displayed a swarm-like behavior with the same characteristics than that of their conspecific males, providing evidence that visual markers are used by the two sexes to join mating spots. Altogether, these results suggest that visual markers and the way species and sexes use them could be key cues in species segregation, swarm location and recognition and consequently in sexual encounter. Our results raise new avenues, first, on the topic of evolution by explaining speciation and diversification within the *An. gambiae* complex and, secondly, on the topic of vector control used for the development of new control tools. For the latter, designing a bio-inspired trap mimicking mating hotspot locations would target both males and females at the same time and could constitute an innovative strategy to reduce vector populations.

## Supplementary information

**Additional file 1: Text S1.** Mosquito colonies, measurement method in the semi-field setup and data analysis**. Figure S1.** Example given for the measurement of the maximal swarm height. **Table S1.** Effects of one-way interactions between marker size, mosquito sex, number of swarming mosquitoes and number of released mosquitoes on swarm characteristics in *An. coluzzii* in the laboratory setup (design 2). **Table S2.** Effects of one- and two-way interactions between marker size, mosquito sex, mosquito species and number of swarming mosquitoes on swarm characteristics in *An. coluzzii* and *An. gambiae* in the semi-field setup. **Table S3.** Effects of one-way interactions between marker size, number of swarming mosquitoes and number of released mosquitoes on swarm characteristics in *An. gambiae* in the laboratory setup (design 2).

## Data Availability

Data supporting the conclusions of this article are included within the article and its additional file. The raw datasets are available from the corresponding author upon reasonable request.

## Abbreviations

GLMM: generalized linear mixed models
L × W × H: length × width × height
