# Supplementary material for: Sex aggregation and species segregation cues in swarming mosquitoes: role of ground visual markers
Source: Parasit Vectors. 2019 Dec 16;12:589. doi: 10.1186/s13071-019-3845-5 (PMC6916054; doi:10.1186/s13071-019-3845-5)
Supplement: Supplementary file 1 — Additional file 1: Text S1. Mosquito colonies, measurement method in the semi-field setup and data analysis. Figure S1. Example given for the measurement of the maximal swarm height. Table S1. Effects of one-way interactions between marker size, mosquito sex, number of swarming mosquitoes and number of released mosquitoes on swarm characteristics in An. coluzzii in the laboratory setup (design 2). Table S2. Effects of one- and two-way interactions between marker size, mosquito sex, mosquito species and number of swarming mosquitoes on swarm characteristics in An. coluzzii and An. gambiae in the semi-field setup. Table S3. Effects of one-way interactions between marker size, number of swarming mosquitoes and number of released mosquitoes on swarm characteristics in An. gambiae in the laboratory setup (design 2). [file 13071_2019_3845_MOESM1_ESM.docx]

**Sex aggregation and species segregation cues in swarming mosquitoes: role of ground visual markers.**

Serge B. Poda^1,2,3^, Charles Nignan^1,2^, Olivier Gnankiné^2^, Roch K. Dabiré^1^, Abdoulaye Diabaté^1^ and Olivier Roux^1,3,*^

1. Institut de Recherche en Sciences de la Santé (IRSS), Bobo-Dioulasso, Burkina Faso.
2. Laboratoire d’Entomologie Fondamentale et Appliquée, Unité de Formation et de Recherche en Sciences de la Vie et de la Terre (UFR-SVT), Université Ouaga I Pr. Joseph KI-ZERBO, Ouagadougou, Burkina Faso.
3. MIVEGEC, IRD, CNRS, University of Montpellier, Montpellier, France.

*Corresponding author: IRD, MIVEGEC, 911 Avenue Agropolis, BP 64501, 34394, Montpellier Cedex 5, France. olivier.roux@ird.fr

**Additional file 1: Text S1. Supplementary methods**

**Mosquito colonies**

The *An. gambiae* colony was established in 2015. *An. gambiae* gravid females were collected in inhabited human dwellings in Soumousso (11°00’46”N, 4°02’45”W), a typical Guinean savannah village located 30 km north-east of Bobo-Dioulasso south-western Burkina Faso, and *An. coluzzii* were collected in Bama (11°24”N; 04°24”W), a village located 30 km north of Bobo-Dioulasso and surrounded by 1,200 ha of irrigated rice fields. Females were placed individually in oviposition cups containing tap water. After oviposition, females were identified according to their species by routine PCR-RFLP based on segregating SNP polymorphisms in the X-linked ribosomal DNA intergenic spacer region [1]. The larvae were gathered according to their species and reared in tap water, fed with Tetramin® Baby Fish Food (Tetrawerke, Melle, Germany) *ad libitum*. Adult mosquitoes were held in 30x30x30cm mesh-covered cages and provided with a 5% glucose solution *ad libitum*. Females were blood fed on rabbits for egg production. Insectarium conditions were 27±2°C, 70±10% relative humidity, 12:12 L:D.

**Experimental setups**

**Measurement method in the semi-field setup**

Measurements smaller than oH (observer size) were read directly on graduated adhesive tapes applied on the compartment wall. All measurements higher than oH were calculated as shown in Figure S1.

**Figure S1. Example given for the measurement of the maximal swarm height (H).**

mH: measured height; oH: height of the observer’s eyes; AB: distance from the observer to the graduate tape; DE: swarm-graduation distance; The red line is the laser used by the observer to read mH; Dotted lines: unknown distances; Full lines: known distances; The height H was calculated using the Thales' theorem.

**Data analysis**

All analyses were performed using R (version 3.4.0) using Generalized Linear-Mixed Models (GLMM, lme4 package) with the appropriate distribution family. The relative location of the swarm as a function of a visual marker was analyzed with a Gaussian distribution. Marker location (labeled 1-6 and 1-3 in the laboratory and semi-field experiments, respectively), species (2 levels: *An. coluzzii* and *An. gambiae*) and sex (2 levels: males and females), and their interactions were considered fixed effects. Mosquito batches (*i.e*. replicates) were considered random effects. The effect of marker size on marker-swarm distance in *An. gambiae* was also analyzed with a subset of data obtained in the semi-field setup with a Gaussian distribution. Marker size, sex, number of swarming mosquitoes and their interactions were considered fixed effect and mosquito batches were considered random factor.

As the protocols used in the laboratory study for marker size effects (design 2) were different for *An. coluzzii* and *An. gambiae* (*i.e.* location of the marker inside or outside the cage, respectively), different species subsets were used in analysis. Similarly, the protocol used to observe *An. gambiae* females was different and was analyzed separately from their respective males. Consequently, in *An. coluzzii* (both sexes) and *An. gambiae* males only, minimal and maximal height, height of the nucleus, width and amplitude were analyzed with a Gaussian distribution. Marker size (3 levels: absence (in *An. gambiae* only), 20 cm and 60 cm), swarm size (*i.e.* estimated number of mosquitoes in the swarm), sex, species (2 levels: in semi-field experiments only) and their interactions were considered fixed effects, and mosquito batches were considered random effects. Swarm size was analyzed using a negative binomial. Marker size, number of mosquitoes in the cage, sexes, species (in semi-field experiments only) and their interactions were considered fixed effects, and mosquito batches were considered random effects.

In *An. gambiae* females (laboratory experiment only, since the swarming behavior was different, see “results”), nucleus height and swarm size were analyzed as described above. Frequency of swarming events (*i.e.* number of events in which one or several females exhibited a swarm-like behavior over the 5min timeframe) was analyzed using a Gaussian distribution. Marker size, number of mosquitoes in the cage and their interactions were considered fixed effects, and mosquito batches were considered random effects.

The effect of insemination on female swarm size and the frequency of swarming events was analyzed with a negative binomial and a Poisson distribution, respectively. Female physiological status (2 levels: virgin and inseminated), number of mosquitoes in the cage and their interactions were considered fixed effects, and mosquito batches were considered random effects. Insemination rates in female populations and in swarming females were compared using a binomial GLMM with mosquito batches as random effects.

For model selection, we used the stepwise removal of terms, followed by likelihood ratio tests. Term removals that significantly reduced explanatory power (*P*<0.05) were retained in the minimal adequate model. Minimal model validations were performed trough analyses of residuals for normality and homogeneity of variances with a Shapiro and Fligner test, respectively. If necessary, data were transformed using the Box-Cox power transformation method (powerTransform, "car" package). All means are provided with their standard error and percentages are expressed with their 95% confident interval.

**Results**

**Table S1.** Effects of one way interactions between marker size, mosquito sex, number of swarming mosquitoes and number of released mosquitoes on swarm characteristics in *An. coluzzii* in the laboratory setup (design 2).

|  | |  | **Marker size x Mosquito Sex** | | |  | **Marker size x Number of swarming mosquitoes** | | |  | **Marker size x Number of released mosquitoes** | | |  | **Mosquito sex x Number of swarming mosquitoes** | | |  | **Mosquito sex x Number of released mosquitoes** | | |
| --- | --- | --- | --- | --- | --- | --- | --- | --- | --- | --- | --- | --- | --- | --- | --- | --- | --- | --- | --- | --- | --- |
|  |  |  | **x²** | **df** | **P-value** |  | **x²** | **df** | **P-value** |  | **x²** | **df** | **P-value** |  | **x²** | **df** | **P-value** |  | **x²** | **df** | **P-value** |
| **Swarm dimensions** | |  |  |  |  |  |  |  |  |  |  |  |  |  |  |  |  |  |  |  |  |
|  | **Maximal** |  | 2.16 | 1 | 0.141 |  | 0.07 | 1 | 0.790 |  | - | - | - |  | 10.23 | 1 | **0.001** |  | - | - | - |
|  | **Nucleus** |  | 0.004 | 1 | 0.944 |  | 0.19 | 1 | 0.660 |  | - | - | - |  | 2.80 | 1 | 0.093 |  | - | - | - |
|  | **Minimal** |  | 0.26 | 1 | 0.605 |  | 3.90 | 1 | **0.048** |  | - | - | **-** |  | 0.36 | 1 | 0.550 |  | - | - | - |
|  | **Amplitude** |  | 0.14 | 1 | 0.703 |  | 0.05 | 1 | 0.810 |  | - | - | - |  | 5.58 | 1 | **0.018** |  | - | - | - |
|  | **Width** |  | 0.94 | 1 | 0.332 |  | 0.002 | 1 | 0.960 |  | - | - | - |  | 11.78 | 1 | **<0.001** |  | - | - | - |
| **Swarm size^a^** | |  |  |  |  |  |  |  |  |  |  |  |  |  |  |  |  |  |  |  |  |
|  | **Number of swarming mosquitoes** |  | 23.39 | 1 | **<0.001** |  | - | - | - |  | 0.87 | 1 | 0.348 |  | - | - | - |  | 0.05 | 1 | 0.817 |

^a^ Swarm size expressed as the number of swarming mosquitoes; Significant P-value are in bold; se: standard error; - : not included in the analyses.

**Table S2.** Effects of one and two way interactions between marker size, mosquito sex, mosquito species and number of swarming mosquitoes on swarm characteristics in *An. coluzzii* and *An. gambiae* in the semi-field setup.

|  |  | **Swarm dimensions** | | | | | | | | | | | | | | | | | | |  | **Swarm size^a^** | | |
| --- | --- | --- | --- | --- | --- | --- | --- | --- | --- | --- | --- | --- | --- | --- | --- | --- | --- | --- | --- | --- | --- | --- | --- | --- |
|  |  | **Maximal** | | |  | **Nucleus** | | |  | **Minimal** | | |  | **Amplitudes** | | |  | **Width** | | |  | **Number of swarming**  **mosquitoes** | | |
| **Interactions** |  | **x²** | **df** | **P-value** |  | **x²** | **df** | **P-value** |  | **x²** | **df** | **P-value** |  | **x²** | **df** | **P-value** |  | **x²** | **df** | **P-value** |  | **x²** | **df** | **P-value** |
| **Marker size x Sex** |  | 1.217 | 1 | 0.27 |  | 0.408 | 1 | 0.52 |  | 0.016 | 1 | 0.90 |  | 0.0002 | 1 | 0.99 |  | 0.197 | 1 | 0.65 |  | 0.542 | 1 | 0.46 |
| **Marker size x Species** |  | 1.205 | 1 | 0.27 |  | 12.348 | 1 | **<0.001** |  | 0.235 | 1 | 0.62 |  | 0.446 | 1 | 0.50 |  | 4.794 | 1 | **0.03** |  | 0.034 | 1 | 0.85 |
| **Marker size x Number of swarming mosquitoes** |  | 2.677 | 1 | 0.10 |  | 0.626 | 1 | 0.43 |  | 0.322 | 1 | 0.57 |  | 0.289 | 1 | 0.59 |  | 0.002 | 1 | 0.96 |  | - | - | - |
| **Species x Sex** |  | 4.987 | 1 | **0.03** |  | 3.674 | 1 | **0.05** |  | 2.424 | 1 | 0.12 |  | 0.403 | 1 | 0.53 |  | 0.727 | 1 | 0.39 |  | 2.122 | 1 | 0.14 |
| **Species x Number of swarming mosquitoes** |  | 0.579 | 1 | 0.44 |  | 1.583 | 1 | 0.20 |  | 3.363 | 1 | 0.07 |  | 2.349 | 1 | 0.13 |  | 0.038 | 1 | 0.84 |  | - | - | - |
| **Sex x Number of swarming mosquitoes** |  | 0.394 | 1 | 0.53 |  | 0.252 | 1 | 0.62 |  | 0.235 | 1 | 0.63 |  | 0.842 | 1 | 0.36 |  | 5.648 | 1 | **0.02** |  | - | - | - |
| **Marker size x Sex x Number of swarming mosq.** |  | 1.383 | 1 | 0.24 |  | 1.355 | 1 | 0.24 |  | 0.0001 | 1 | 0.99 |  | 0.520 | 1 | 0.47 |  | 1.233 | 1 | 0.26 |  | - | - | - |
| **Marker size x Species x Number of swarming mosq.** |  | 0.305 | 1 | 0.58 |  | 3.457 | 1 | 0.06 |  | 1.473 | 1 | 0.23 |  | 2.874 | 1 | 0.09 |  | 0.513 | 1 | 0.47 |  | - | - | - |
| **Marker size x Species x Sex** |  | 6.068 | 1 | **0.01** |  | 2.811 | 1 | 0.09 |  | 1.844 | 1 | 0.17 |  | 0.519 | 1 | 0.47 |  | 0.916 | 1 | 0.34 |  | 0.124 | 1 | 0.72 |
| **Species x Sex x Number of swarming mosquitoes** |  | 0.018 | 1 | 0.89 |  | 2.504 | 1 | 0.11 |  | 2.424 | 1 | 0.12 |  | 1.707 | 1 | 0.19 |  | 0.358 | 1 | 0.55 |  | - | - | - |

^a^ Swarm size expressed as the number of swarming mosquitoes; Significant P-value are in bold; se: standard error; - : not included in the analyses.

**Table S3.** Effects of one way interactions between marker size, number of swarming mosquitoes and number of released mosquitoes on swarm characteristics in *An. gambiae* in the laboratory setup (design 2).

|  |  | |  | **Marker size x Number of swarming mosquitoes** | | |  | **Marker size x Number of released mosquitoes** | | |
| --- | --- | --- | --- | --- | --- | --- | --- | --- | --- | --- |
|  |  |  |  | **x²** | **df** | **P-value** |  | **x²** | **df** | **P-value** |
| **Males** | **Swarm dimensions** | | |  |  |  |  |  |  |  |
|  |  | **Maximal** |  | 0.13 | 2 | 0.936 |  | - | - | - |
|  |  | **Nucleus** |  | 2.35 | 2 | 0.312 |  | - | - | - |
|  |  | **Minimal** |  | 0.17 | 2 | 0.922 |  | - | - | - |
|  |  | **Amplitude** |  | 0.17 | 2 | 0.911 |  | - | - | - |
|  |  | **Width** |  | 5.91 | 2 | 0.053 |  | - | - | - |
|  | **Swarm size^a^** | |  |  |  |  |  |  |  |  |
|  |  | **Number of swarming mosquitoes** |  | - | - | - |  | 3.08 | 2 | 0.211 |
| **Females** | **Swarm dimensions (±es) cm** | |  |  |  |  |  |  |  |  |
|  |  | **Height** |  | 5.55 | 2 | 0.062 |  | - | - | - |
|  | **Swarm size** | |  |  |  |  |  |  |  |  |
|  |  | **Number of swarming mosquitoes** |  | - | - | - |  | 0.41 | 2 | 0.822 |
|  | **Swarming frequency** | |  |  |  |  |  |  |  |  |
|  |  | **Frequency** |  | - | - | - |  | 2.11 | 2 | 0.351 |

^a^ Swarm size expressed as the number of swarming mosquitoes; se: standard error; - : not included in the analyses.

**References**

1. Santolamazza F, Mancini E, Simard F, Qi Y, Tu Z, Della Torre A. Insertion polymorphisms of SINE200 retrotransposons within speciation islands of *Anopheles gambiae* molecular forms. Malar. J. 2008;7:163.
